# Determination of regional lung air volume distribution at mid-tidal breathing from computed tomography: a retrospective study of normal variability and reproducibility

**DOI:** 10.1186/1471-2342-14-25

**Published:** 2014-07-25

**Authors:** John Fleming, Joy Conway, Caroline Majoral, Michael Bennett, Georges Caillibotte, Spyridon Montesantos, Ira Katz

**Affiliations:** 1 National Institute of Health Research Biomedical Research Unit in Respiratory Disease, University Hospital Southampton NHS Foundation Trust, Southampton, UK; 2 Department of Medical Physics and Bioengineering, University Hospital Southampton NHS Foundation Trust, Southampton, UK; 3 Faculty of Health Sciences, University of Southampton, Southampton, UK; 4 Medical R&D, Air Liquide Santé International, Centre de Recherche Claude-Delorme, Les Loges-en-Josas, France; 5 Department of Mechanical Engineering, Lafayette College, Easton, PA, USA; 6 Department of Nuclear Medicine, Southampton General Hospital, Mail Point 26, Southampton SO166YD, UK

**Keywords:** Regional lung volume measurement, Computed tomography, Reproducibility

## Abstract

**Background:**

Determination of regional lung air volume has several clinical applications. This study investigates the use of mid-tidal breathing CT scans to provide regional lung volume data.

**Methods:**

Low resolution CT scans of the thorax were obtained during tidal breathing in 11 healthy control male subjects, each on two separate occasions. A 3D map of air volume was derived, and total lung volume calculated. The regional distribution of air volume from centre to periphery of the lung was analysed using a radial transform and also using one dimensional profiles in three orthogonal directions.

**Results:**

The total air volumes for the right and left lungs were 1035 +/− 280 ml and 864 +/− 315 ml, respectively (mean and SD). The corresponding fractional air volume concentrations (FAVC) were 0.680 +/− 0.044 and 0.658 +/− 0.062. All differences between the right and left lung were highly significant (p < 0.0001). The coefficients of variation of repeated measurement of right and left lung air volumes and FAVC were 6.5% and 6.9% and 2.5% and 3.6%, respectively. FAVC correlated significantly with lung space volume (r = 0.78) (p < 0.005). FAVC increased from the centre towards the periphery of the lung. Central to peripheral ratios were significantly higher for the right (0.100 +/− 0.007 SD) than the left (0.089 +/− 0.013 SD) (p < 0.0001).

**Conclusion:**

A technique for measuring the distribution of air volume in the lung at mid-tidal breathing is described. Mean values and reproducibility are described for healthy male control subjects. Fractional air volume concentration is shown to increase with lung size.

## Background

X-ray computed tomography (CT) is excellently suited to visualisation and accurate measurement of air volume in the airway tree [1]. CT scanning provides maps of the linear attenuation coefficient of x-rays at an average energy of 70 kV. At this energy the attenuation coefficients of low atomic number tissues such as soft tissue are closely proportional to density. Thus CT images of the lung reflect density and therefore have the advantage of providing accurate values of the air volume in the lung. They give images of the regional distribution of air volume in addition to enabling values of total lung volume to be assessed. With multi-detector high resolution CT, the whole lung field can be imaged in less than 10 s enabling images to be obtained while subjects hold their breath at a particular level of respiration. Images at total lung capacity are most common, but images at other levels of respiration can be acquired [2]. Image analysis of the CT data allows segmentation of approximately the first six generations of the airway tree, enabling accurate definition of the geometry of this section of the airway [3]. This information can be applied to derive functional parameters using computational fluid dynamics [4]. While the peripheral and alveolated airways are too small to be visualised individually, the pattern of their appearance in the images can be used to characterise typical patterns occurring in different diseases. A simple example is the assessment of the density of the peripheral area to give an emphysema score [5]. More complex pattern recognition algorithms can be used to identify the appearance of different diseases [6]. In particular, a method involving parametric response maps derived from CT data acquired at full inspiration and full expiration has been described, which is useful in distinguishing between emphysema and functional small airways disease [7].

CT-derived parameters have also been used to follow developmental and compensatory lung growth [8] and to assess structural changes in idiopathic pulmonary fibrosis [9] and emphysema [10].

Another area where measurements of regional lung volume are used is in normalisation of measurements of the regional distribution of aerosol deposition using radionuclide imaging. The relative deposition in conducting and peripheral airways is often assessed by a central to peripheral ratio. The values obtained depend quite significantly on the size of the areas chosen. This dependency can be considerably reduced by normalising the central to peripheral ratios to lung volume [11,12]. Lung volume is often estimated using transmission scanning or ventilation imaging. Both methods have disadvantages – delineation of the lungs on transmission is imprecise and ventilation does not reflect lung volume in poorly ventilated areas. The ability to use CT for this purpose overcomes these limitations. The disadvantages of CT are its relatively high radiation dose and its accessibility. Dose may be reduced using low dose protocols [13] and the use of CT in conjunction with both gamma camera tomography (Single Photon Emission Computed Tomography, SPECT) and positron emission tomography (PET) is becoming increasingly commonplace.

Measurements of lung anatomy from CT have a further application in the field of inhaled aerosol deposition. They provide anatomical information which allows improved computer modelling of the deposition pattern. In this respect images of lung volume obtained at mid-tidal breathing such as those obtained in some SPECT-CT protocols are particularly useful in that they provide information on lung anatomy at the same stage of breathing as is frequently used in experiments on aerosol inhalation.

An important disadvantage of CT, and indeed a fundamental problem of most three-dimensional imaging studies, is that the images are acquired in the horizontal position. It is known that lung anatomy varies very significantly between supine and erect positions; the FRC of the lung is greater by around 40% with the subject erect compared to supine [13]. Therefore all information regarding lung anatomy including lung volume values have to be compared in the light of this large effect.

In addition, it has been shown that even when volume measurements in the supine position are compared, there are significant differences between values from CT and other methods, with CT values being generally lower than other techniques [14-16]. While CT scanning is now mentioned in standard texts on lung volume assessment [1], there is a shortage of reference values for expected lung volume values in control subjects. Reproducibility data is available for CT lung volumes obtained at total lung capacity and residual volume [17]. Also, some work has been done on reproducibility of lung volume values from CT in preclinical studies [13].

In this study measurements of lung volume have been made using low resolution (and therefore low dose) CT during tidal breathing in 11 healthy subjects in the supine position. This has enabled mean values of total lung volume and patterns of regional distribution to be obtained at a mid-tidal breathing position, together with measures of inter-subject variability. Each subject was imaged on two separate occasions enabling the intra-subject reproducibility of the measurements to be assessed. The results presented in this paper are derived from data acquired in a previously described experiment [18]. The prime purpose of that work was to provide data for validation of computer models of aerosol deposition by comparison with experimental measurements using medical imaging. The information on lung volume described herein is a by-product derived from that image dataset.

## Methods

### Subjects

The subjects described in this paper were recruited for a project to validate computer predictions of aerosol deposition by comparison with experimental measurements using medical imaging [18]. Eleven healthy, never-smoker, male subjects, between 20 and 45 years old were studied. Subjects had no evidence of respiratory disease and lung function tests within the normal range. This included being free from the common cold and rhinitis for at least four weeks before entry into the study. The study was approved by the Local Research Ethics Committee of Southampton University Hospital NHS Trust and the Administration of Radioactive Substances Advisory Committee, and patients gave written consent to participate in it. The nature of the study was explained to the subjects and each one signed a consent form. Measurements were made of height, weight, FEV1 and FRC. These are shown in Table 1.

**Table 1:** **Subject characteristics: height, weight, FEV1**_**1 **_**and FRC**

**Subject**	**Height (cm)**	**Weight (kg)**	**FEV**_**1 **_**(l)**	**FRC ml**
P00	185	91	4.82	3080
P01	180	83	5.24	3170
P02	174	56	4.27	3370
P03	171	75	4.68	3343
P04	173	80	3.88	2800
H01	174	82	4.85	2360
H02	187	88	5.57	4800
H03	177	78	4.27	3170
H04	186	87	5.48	4170
H05	173	80	3.76	3480
H06	179	N/A	4.98	2910

### Acquisition of the CT scans

The CT scans were acquired as part of a combined SPECT-CT protocol on a GE Infinia dual head gamma camera with the Hawkeye 4 CT attachment (GE Medical Systems, Milwaukee, Wisconsin, USA). The participants were placed supine on the couch with the whole lung field included in the field of view. The CT scan consisted of 90 slices with an interslice separation of 4.42 mm. It used a voltage of 120 kVp and a current of 1.0 mA. The image took approximately 4 mins to acquire and therefore provided a CT image of the thorax at mean tidal breathing. The effective dose received by the subjects from the CT procedure was 0.8 mSv. The CT images have a resolution of the order of 1 mm in the transaxial plane, but this is limited in the axial (superior-inferior) direction by the inter-slice separation of 4.42 mm. The resolution in all dimensions is blurred, as the image is obtained as an average over the tidal breathing cycle.

### Segmentation of the lung space

The CT images were transferred to a workstation running the Portable Imaging Computer software (PICS) processing system [19]. The analysis was carried out using a fully three dimensional approach and therefore the first step was to convert the data to 4.42 mm cubic voxels. It was next necessary to delineate the outer boundary of the lung envelope. This is straightforward in principle in that lung voxels are generally easily separated from those containing tissue by virtue of their different density and therefore different Hounsfield number. However in practice the algorithms to delineate the lung envelope are not straightforward [20,21]. In this study a semi-automatic approach has been applied; the method has been described in detail previously [22] and a brief outline is presented here.

**Figure 1:**
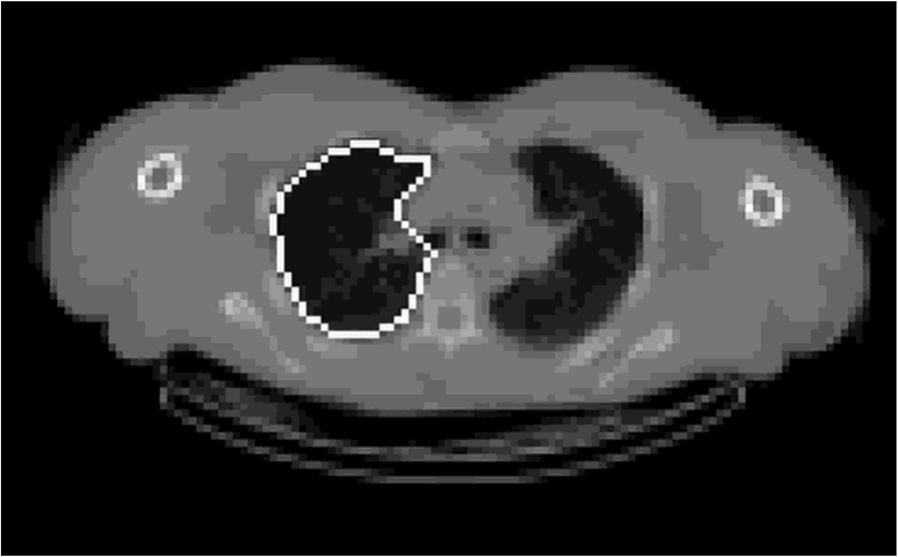
**Example of the final segmentation of the right lung in the region of the right hilum.** The outline remains close to the lung edge around the lung periphery and includes the vessels present in the area of the hilum.

The positions of a seed point in the trachea and the hila of the right and left lungs were defined manually from an interactive visual display of the images. The hilum was taken as the point of the first bifurcation of the main bronchus, which was readily visible from a coronal view for the right lung or a transaxial view for the left. The software then proceeded automatically to define regions for each lung and the trachea/main bronchi using a threshold-based technique [22]. A typical segmentation of the right lung from the CT image in a transverse slice is illustrated in Figure 1.

### Formation of the lung volume image

**Figure 2:**
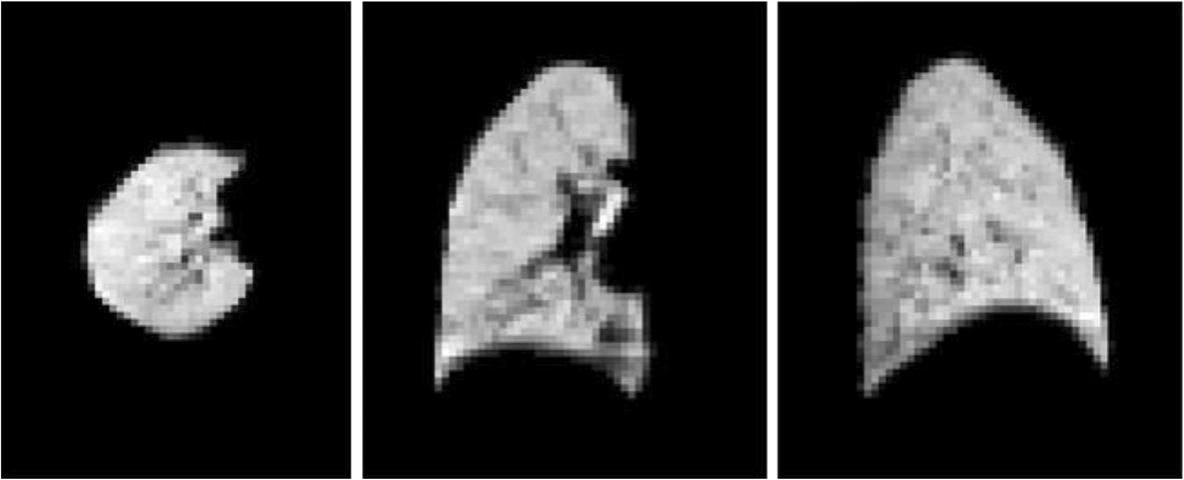
Example 2D transverse, coronal and sagittal sections from a 3D volume image obtained from a CT scan.

X-ray CT images consist of a map of attenuation coefficients for photons produced by the x-ray tube. For the energy used in CT, the attenuation coefficient for the soft tissue voxels in the lung can be assumed to be proportional to density. Therefore the density of the voxel, ρ_v_, can be calculated using the following equation:

(1) $$\rho_{v}=\frac{I_{\mathrm{vox}}-I_{\mathrm{air}}}{I_{\mathrm{tissue}}-I_{\mathrm{air}}}$$

where I_air,_ I_tissue_ and I_vox_ are the image intensity values for air, tissue and the voxel under consideration respectively. The fractional air volume concentration of each voxel, (FAVC) can then be calculated as:

(2) $$\mathrm{FAVC}=\left(1-\frac{\rho_{v}}{\rho_{t}}\right)$$

where ρ_t_ is the average density of the tissue and blood in the lung, which is taken as 1.05 [23]. The air volume in the voxel is then FAVC.v where v is the voxel volume. It is current practice to use density as the descriptive parameter when describing CT scans. However it is considered that this new parameter, FAVC, is more appropriate to use when considering measurements of air volume. The two parameters are simply related with FAVC being approximately one minus the density value. Two dimensional sections through a typical 3D volume image derived from CT are shown in Figure 2.

### Calculation of lung volume

The lung volume is calculated by summing the air volume in all the voxels in the lung envelope [14]. This simple summation will result in a small underestimate of volume due to the partial volume effect. This occurs due to the finite resolution of the images which causes voxels on the boundary to have increased density and hence falsely low estimates of air volume. However this is exactly compensated by voxels just outside the lung having reduced density and therefore some apparent air content. To correct for this effect, the lung envelope was dilated by 1 voxel (4.42 mm) and the summation of lung air volume performed within this dilated volume. Both right and left total lung volumes were calculated.

### Determination of mean volume image

**Figure 3:**
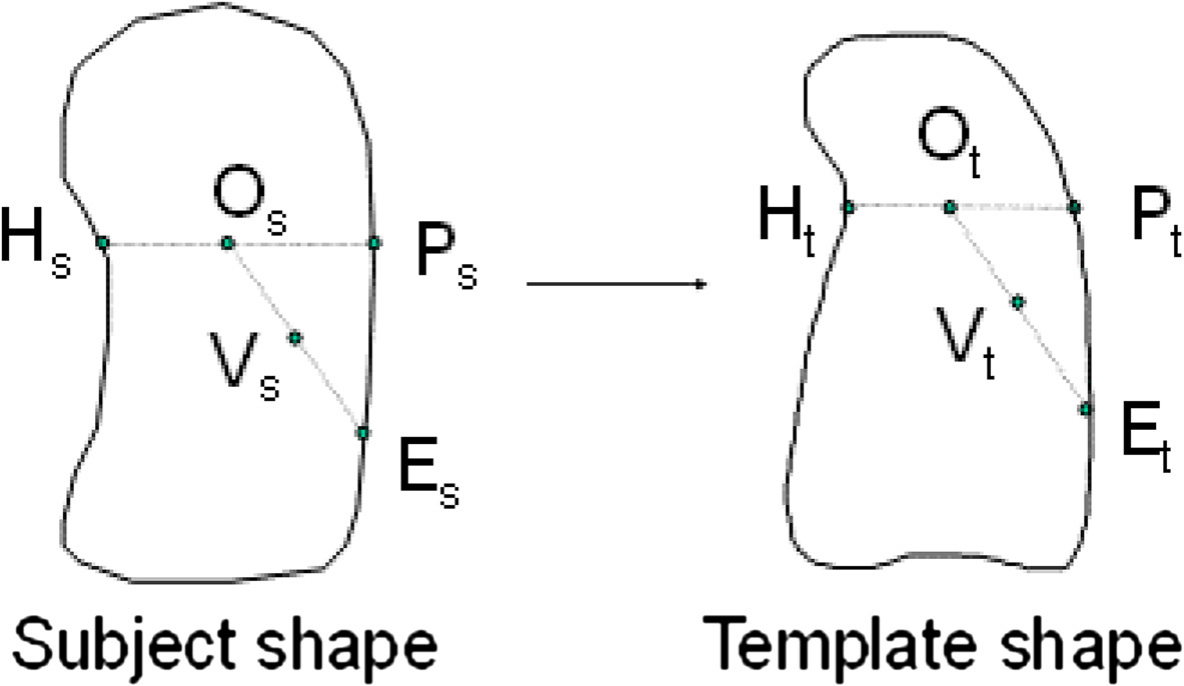
**Schematic diagram illustrating the radial transform method of elastic registration of the images.** The subject’s image of fractional air volume concentration (FAVC) is transformed to the template shape by finding for each voxel in the template shape V_t_. the corresponding voxel in the subject shape V_s_. This is based on the direction cosines and the fractional radial distance of V_t_ relative to the centre of the transform O_t_. The voxel in the template shape, V_t_, is then filled with the FAVC value at V_s_.

It is often valuable to describe the average appearance of a particular organ of the body by forming mean images across a range of subjects. Therefore a mean image of lung volume was created for the male healthy control subjects included in this study. As the lungs in different subjects have different shapes and sizes, it was necessary to register each image to a standard template. One of the subjects with a close to average total lung volume was chosen as the template, and each of the others were registered to the template using a radial transform based on the definition of the lung outline of the subject under investigation and the standard template. This was preferred to more complicated schemes, such as active shape models, for its simplicity. The transform also required definition of a single common anatomical location in both the subject and template. The hilum of the lung is in many respects the natural choice for the centre of a spherical transform, as both airways and blood vessels branch out approximately radially from this point. However, the hilum is either on, or close to, the edge of the lung and therefore cannot be used, as nearly half the radial paths from it have a very short or zero length within the lung. Therefore a point in the centre of the lung O (o_x_, o_y_, o_z_) is chosen, which is related to the hilum position H (h_x_, h_y_, h_z_) such that:

(3) $$o_{x}=\;\left(h_{x}+\;p_{x}\right)\;/2,\;o_{y}\;=\;h_{y},\;o_{z}=\;h_{z}$$

where x, y and z represent the left/right, anterior/posterior and superior/inferior directions respectively. p_x_ is the x position of the lateral edge point on the lung at the same y and z co-ordinates as the hilum. The transform is illustrated in Figure 3. The purpose is to determine, for each voxel in the template shape, the corresponding position in the subject shape and then transfer the value of the CT image in the subject voxel to the equivalent template voxel. Each voxel V_t_ in the template shape is characterised by its direction cosines and the fractional radial distance (f_t_ = OV_t_ / OE_t_) from the central point (O_t_) to the equivalent extrapolated position on the edge (E_t_). The line OE_s_ in the subject shape is defined as that passing through O_s_ with the same direction cosines as OV_t_ . For each voxel along this line the fractional distance to the edge (f_s_ = OV_s_ / OE_s_) is calculated. The voxel, V_s_, with the value of f_s_ nearest to f_t_, is found. The CT image value in this voxel is transferred to voxel, V_t_, in the template shape. This process is repeated for all the template voxels. Prior to registration the images were expressed in terms of the fractional air volume concentration per unit space volume, FAVC, and these absolute values were preserved in the elastic transform process.

The process was applied to the right and left lungs separately for one of the images obtained from each subject and then a combined right and left image produced. These 11 combined images were then averaged on a voxel by voxel basis to produce the mean image of fractional air volume concentrations. As the outer layers of voxels were affected by the partial volume effect resulting in a systematic reduction of the values a correction was applied. Using successive erosion of the volume outline by three voxels the average air volume concentration in each was voxel layer was found. As expected the outer two layers were reduced compared to the third layer. It was then assumed that the average FAVC value in all three layers was the same and therefore all voxels in each of the outer two layers were multiplied by the appropriate factor to equalize the average FAVC.

One dimensional profiles of the average fractional air volume concentration were then derived in three orthogonal directions from the mean volume image.

### Shell analysis of volume

The radial transform concept was also applied to determine the variation of fractional air volume concentration from the centre to the periphery of the airway tree. In this case the transform was carried out using the hilum as the centre of the transform with the lung being divided into ten concentric shells [24]. For each subject the total air volume per shell, V_i_ and mean fractional air volume concentration per shell FAVC_i_ were calculated. The mean and standard deviation fractional air volume concentration per shell across all subjects were calculated and the intra subject reproducibility was assessed. This data was summarized as a central to peripheral ratio defined by

(4) $$c/p=\frac{\sum_{i=1}^{5}V_{i}}{\sum_{i=6}^{10}V_{i}}$$

### Statistical analysis

All systematic differences between lung volume parameters were assessed using the t-test. For comparing parameters between left and right lungs and in intra-subject repeatability assessment, the paired t-test was used. Correlations between parameters were performed using linear correlation. Random differences in intra-subject variability were assessed from the standard error of the estimate of linear regression between the two sets of measurements, expressed as a percentage of the mean. Comparisons between measures of variability were performed using the F-test. The statistical analyses were performed using Microsoft Excel (Redmond, WA).

## Results

### Total lung volume parameters

**Figure 4:**
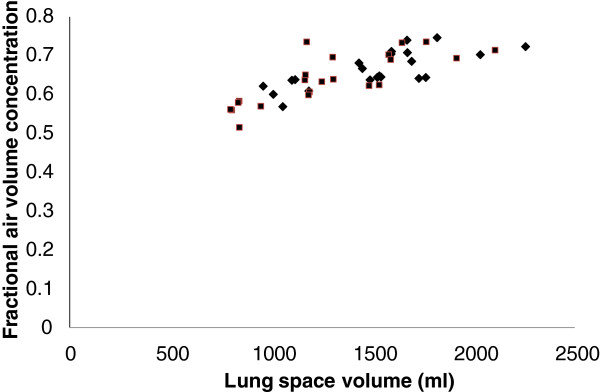
The variation of fraction air volume concentration with lung space volume in control subjects for the right (♦) and left (■) lungs.

**Figure 5:**
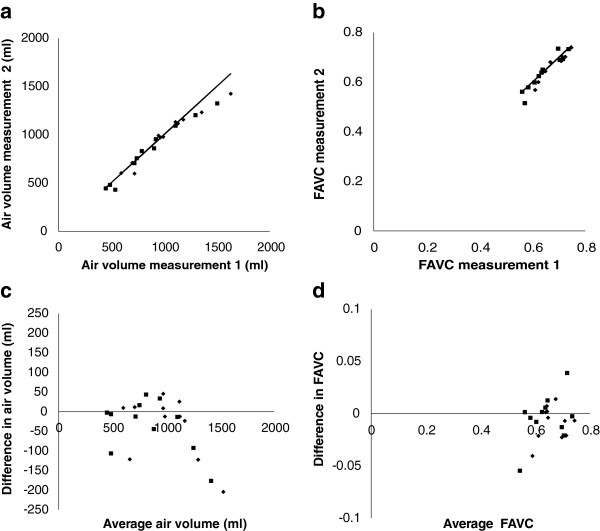
**This illustrates the reproducibility of measuring parameters of lung volume.** Graph **(a)** shows the results for total volume and **(b)** for fractional air volume concentration (FAVC) for the right lung (♦) and left lung (■). In each graph, the line is the line of identity. The corresponding Bland-Altman plots of difference in measurements against mean of measurements are shown in graphs **(c)** and **(d)**.

The results of measurements of space volume, air volume, and fractional air volume concentration are summarized in Table 2. The mean values of all parameters were significantly greater for the right lung than for the left (p < 0.0001). The coefficient of variation of inter-subject variability of air volume was significantly greater than for the air volume concentration (p < 0.01). The coefficients of inter-subject variability for all parameters were greater for the left lung compared to the right, but in no case were the numbers sufficient to give statistical significance.The total air volume in both lungs from CT correlated significantly with an erect measurement of FRC (r = 0.67, p < 0.01, COV 15%). The mean total volume (1900 ml) was significantly lower than for FRC (3332 ml). The supine volume from CT was therefore 43% reduced from the erect value obtained by helium dilution. The inter-subject coefficient of variation for FRC (19.9%) was lower than that for CT volume (31.6%) but this difference just failed to achieve significance. Fractional air volume concentration correlated significantly with space volume (r = 0.78, p < 0.005) (Figure 4).The coefficients of variation of repeated measurement of right and left lung space volumes and air volumes were 5.3% and 5.8%, and 6.5% and 6.9%, respectively. The correlation of repeat measurements of lung volume for both lungs is shown in Figure 5a. The corresponding Bland-Altman plot, showing the variation of the difference between the measurements and their mean is shown in Figure 5c. This suggests that the repeatability in terms of absolute volume may increase with lung size but more subjects would be required to confirm this. The coefficients of variation of intra-subject repeatability for the mean fractional air volume concentration were 2.5% and 3.6%, respectively. The correlation of repeat measurements is shown in Figure 5b and the corresponding Bland-Altman plot in Figure 5d.

**Table 2:** Summary of results of measuring global values of lung volume and fractional air volume concentration

	**Mean (ml)**	**Standard deviation (ml)**	**Coefficient of variation (%)**	**Coefficient of variation of intra subject reproducibility (%)**
Right lung space volume	1506	334	22.2	5.3
Left lung space volume	1208	375	31.0	5.8
Right lung air volume	1035	280	27.1	6.5
Left lung air volume	864	315	36.5	6.9
Fractional air volume concentration right	0.680	0.044	6.5	2.5
Fractional air volume concentration left	0.658	0.062	9.4	3.6

### Regional lung volume parameters

**Figure 6:**
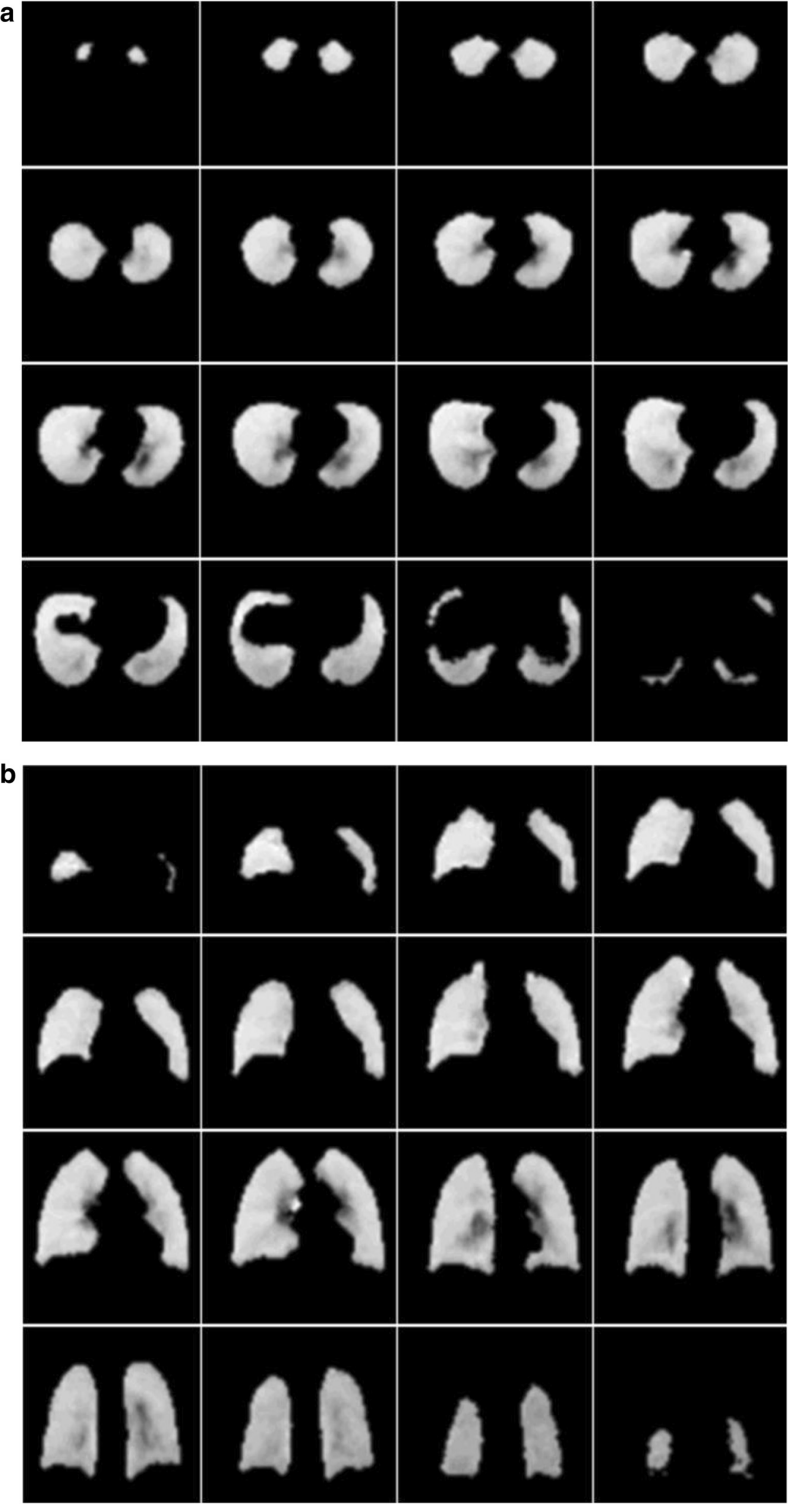
**This illustrates the mean 3D air volume concentration dataset.** Image set **(a)** shows transverse slices running from apex to base of the lung and image set **(b)** coronal slices running from anterior to posterior.

**Figure 7:**
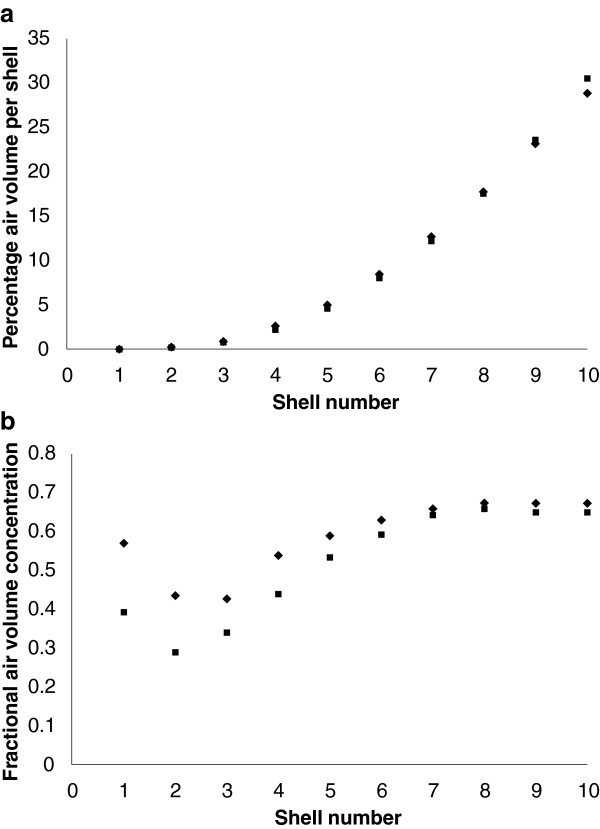
**This illustrates the results of shell analysis of lung air volume.** The graphs show the variation of **(a)** the percentage of total lung air volume and **(b)** fractional air volume concentration from the centre of the lung (shell 1) to the periphery (shell 10). Data for the right (♦) and left (■) lungs are shown separately.

Transverse and coronal slices sampled through the mean air volume concentration images are shown in Figure 6. The full mean volume data is available on the departmental website. The results of shell analysis of the air volume data are shown in Figure 7. This illustrates the variation of mean total air volume and mean fractional air volume concentration for both the right and left lungs. The fractional concentration of air in shell 1 assumes an intermediate value for both lungs. Since the origin of the radial transform is at the hilum, which is taken as the centre of the airway where the main bronchus bifurcates, some of the voxels of shell one will contain air, while others will contain the airway wall surrounding the bronchus. These balance out to give an air volume concentration of around 0.5. The air volume concentration then decreases with shell number due to the presence of major blood vessels in shells 2 and 3. The fraction of air then increases with shell number reaching a fairly constant mean value of about 0.68 in the outer 4 shells. The concentration is slightly higher for the right lung (0.69) than for the left (0.67).

**Figure 8:**
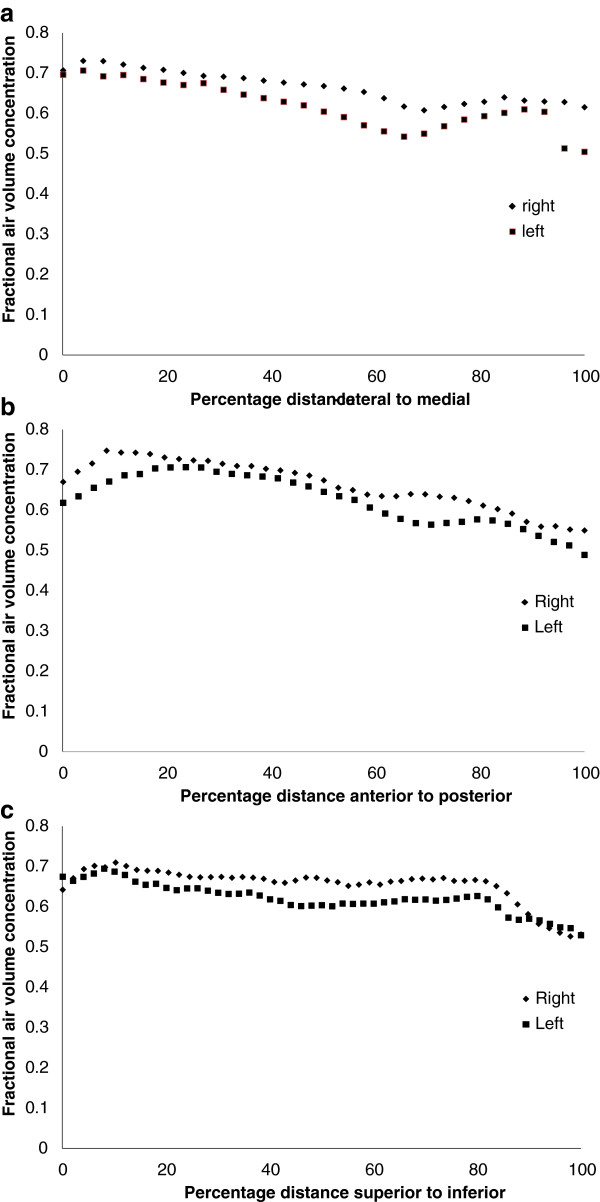
Variation of fractional air volume concentration within the lung envelope (a) from lateral to medial position, (b) from anterior to posterior position, and (c) from superior to inferior position.

The mean value of the central to peripheral (c/p) ratio of air volume for the left lung of (0.089 +/− 0.013 SD) was significantly less than that of the right lung (0.100 +/− 0.007 SD) (p < 0.0001). The coefficient of variation of intra-subject reproducibility for repeated measurements of the c/p ratio was 8.5%.The results of forming one dimensional profiles of the mean volume data set are shown in Figure 8. Figure 8a shows the variation of air volume concentration within the lung envelope from lateral to medial position. This demonstrates the decrease of air concentration medially due to the presence of the large blood vessels in the medial region. Figure 8b shows the variation of air volume concentration within the lung envelope from anterior to posterior position. This demonstrates the decrease of air concentration posteriorly, presumably due to the effect of gravity in the supine position. Figure 8c shows the variation of air volume concentration within the lung envelope from superior to inferior position. There is a general decrease from superior to inferior. This may be due to compression of the lower parts of the lung in the supine position due to pressure from the abdomen contents. The more rapid decrease at the lung base could be an artefact of the partial volume effect. Although a correction for the partial volume effect has been applied this is only on the basis of the average effect over the whole outer voxel layer. The lung sections at the base become very narrow, and are therefore more affected by the partial volume effect than the average for the lung.

## Discussion

This study has demonstrated that it is possible to use low resolution, and therefore low dose CT, to provide quantitative images of lung volume at approximately mid-tidal breathing. These depict the fractional lung air volume per unit space volume and they enable, among other things, the total air volume of the lungs to be obtained. A normal range for this has been defined in a set of 11 subjects and an assessment of intra-subject variability between repeat measurements has been obtained. A method for producing a mean lung volume map has been described and used to provide such a map for this group of subjects. This has been analysed to produce a description of the regional variation of fractional air volume from (i) centre to periphery of the lung using a shell analysis and (ii) as air volume profiles in three orthogonal directions. The reproducibility of the central to peripheral ratio of air volume derived from the shell analysis has also been measured.

The methodology described in this paper is not fully automated, and therefore not ideal for routine use. However algorithms are now commercially available, e.g. from VIDA Diagnostics, USA, which are capable of fully automatic segmentation of the lung and determination of lung volume. It will be interesting to see whether this software is capable of being used with low dose images such as those described in this paper and also whether the values obtained of lung volume at mid tidal breathing are similar. A key aspect of the segmentation of the lung and the subsequent analysis is the definition of the position of hilum of the lung and how much of the area around the hilum is included in the lung envelope. In this paper we have used the first bifurcation of the main bronchus to define the hilum and have used a technique involving dilation and erosion to fill in the areas in the lung envelope with high density including those around the hilum. It would be useful for the lung imaging community to make an effort to standardise these definitions so that results of different software will be comparable.

The results on total lung volume are now considered. As expected, the right lung space volume was found to be significantly higher than the left lung, contributing 53.9% of the total space volume of the lungs. The average fractional air volume concentration was also significantly higher for the right lung with the result that the fraction of air volume from the right lung is slightly but significantly higher at 54.8%. Consistent with these findings, the averaged coefficient of inter-subject variability was found to be higher for the air volume than for the space volume, 32% compared to 27%. The inter-subject variability of fractional air volume concentration was considerably lower at 8.6%.

The average fractional air volume concentration in each lung correlated significantly with the total space volume of the lung. This is in agreement with the study by Gevenois et al. [25], who observed the fractional air volume at full inspiration in the supine position correlated with total lung capacity in healthy volunteers. This correlation may be explained by considering the gas exchange requirements of the lung. If density were to remain the same as body size increases then both the space volume and the air volume of the lung would increase proportionately. Assuming that alveolar volume is the largest component of lung volume, the volume of alveoli will then increase in proportion to body size. However, the surface area available for gas exchange will only increase proportional to body size to the power two thirds. Therefore to allow for this, the alveolar volume will have to increase at a rate which is greater than proportional to body size to maintain the required increase in alveolar surface area. It should also be noted that the difference in FAVC between left and right lungs also followed this pattern with the larger right lung also having the higher FAVC. This variation will be important to bear in bind when interpreting results of density measurements in the assessment of emphysema. Since most diagnostic CT scanning is carried out at full inspiration or full exhalation with high resolution CT, it will be important to confirm this finding in subjects imaged at these states of inhalation.

The mean total, (i.e. left plus right), mid-tidal air volume in the supine position used for imaging was 1900 ml. This is reduced by 43% from the erect FRC value obtained in these subjects using helium dilution of 3332 ml. Using the standard equation described by Stock and Quanjer [26], the mean predicted erect FRC for these subjects was found to be 3377 ml. This is very close to the mean value found, so this group of subjects can be considered to represent a typical adult male population. Ibanez and Raurich [27], found the average male supine FRC using He dilution to be 2230 ml. Therefore the CT values found in this paper are reduced by 330 ml compared to the helium dilution technique. One reason why the mean value obtained from CT will be lower is that it only describes the air volume from the lung hilum onwards. It does not include the nasopharynx, oropharynx, trachea and main bronchi. All this volume will be included in a helium dilution measurement. The mean total volume of this space in adult males is 226 ml. The components of this are nasopharynx, 138 ml [26], oropharynx, 61 ml [28], trachea, 19 ml [29] and main bronchi 8 ml [30]. This can go some way to explain the lower value obtained with CT. However, this will be balanced by the fact that a CT image obtained during tidal breathing might be expected to be higher than FRC as it should be equal to FRC plus half the tidal volume. Since the mean tidal volume is around 500 ml this means that CT values might be expected to be around 250 ml higher. This almost exactly balances out the underestimation described above due to the missing extra-pulmonary volume.

The above arguments lead us to consider whether there is any technical reason for an underestimation in the CT measurements. The most likely explanation here is due to the partial volume effect resulting from the limited spatial resolution of the scans. A first order correction for this effect has been applied as described above. However this only uses a global correction to the missing volume close to the surface of the lung and it may not apply accurately to all parts of the lung where local effects may be important. For example towards the base of the lung, the organ becomes very narrow and in some slices there is a thin section of lung volume, where no voxel may come above the threshold. The global correction applied will allow for this to some extent, but it may not be sufficient. This effect will be greater in low resolution CT studies such as those described here, particularly as mean tidal breathing has been used as compared to a breath hold. Investigation of the magnitude of this effect using image simulation techniques will be the subject of further study.

The correlation of CT lung air volume with FRC was reasonably good (COV 15%) and comparable with previously described results [31]. CT imaging might be considered as an alternative method for measuring FRC, although the large difference in supine compared to erect values would need to be taken into consideration in interpreting the results. Further studies comparing CT and helium dilution should ideally measure a supine FRC with helium dilution to enable a direct comparison of results. The inter-subject variability of CT lung air volume (32%) was not significantly different from that of FRC (20%). However it was considerably larger than for FRC. Increased number of subjects will enable clarification of whether this difference is significant.

There was good intra-subject reproducibility for total lung air volume in these control subjects, with a coefficient of variation of repeat measurements on separate occasions being 6.5%. This compares favourably with previous assessments of reproducibility [13]. The intra-subject coefficient of variation of average fractional air volume concentration was even lower at 3.1%. However this reduced variation might be expected given the lower inter-subject variation for this parameter compared to total air volume.

CT scans of the lung give the opportunity of investigating the three dimensional distribution of air volume in the lung at various stages of respiration. Most work to date has centred on looking at the number of voxels with very high air volume concentration at full inspiration as a measure of emphysema [5]. The values obtained for this parameter will be dependent on the precise imaging protocol [32] and analysis of these low resolution scans of the airway tree at average tidal breathing are not likely to be useful for this purpose. The elastic registration of each image to a standard template has enabled a mean image of the distribution of air volume to be obtained. This provides a database of the normal distribution of air volume against which air volume distributions of subjects with suspected lung diseases might be compared using techniques such as statistical parametric mapping [33]. The method of non-rigid registration described in this paper uses a simple global spatial transformation, suitable for the relatively low resolution spatial images acquired. More sophisticated techniques would be required for high resolution images where fiducial markers such as the bifurcation points can be identified using commercially available software (e.g. Apollo, VIDA Diagnostics, USA). These would allow a more precise local deformation to be applied.

Two methods of analysing the spatial distribution of air volume in terms of one-dimensional vectors have been described in this paper. The first is the radial transform or shell analysis technique which allows a description of the variation of air volume concentration from centre to periphery of lungs. The different shells defined in the analysis correlate approximately with airway generation number and therefore provide a first order approximation to distribution of air volume concentration with airway generation. This form of analysis has been used in describing the three dimensional spatial variation of inhaled aerosol deposition from the centre to the periphery of the lung [24]. In both two dimensional [11] and three dimensional [34] imaging of aerosol deposition, it has been found valuable to normalise the shell data to the distribution of either ventilation or lung volume. Normalisation to ventilation has the disadvantage that it may not be valid in diseases of the lung with abnormal ventilation. Normalisation to lung air volume seems a more robust method and, as CT provides the best imaging of lung air volume distribution available, it could be regarded as the gold standard method for this type of normalisation. The finding that the central to peripheral ratio of lung air volume was different for the left and right lungs shows that normalisation of aerosol deposition should be carried out separately for each lung. The intra-subject reproducibility for the c/p ratio of 8.5% was considered to be reasonably good, although more variation was found for this parameter compared to measures of the total air volume in the lung.

The other method of analysis described is the one dimensional analysis of fractional air volume concentration in three orthogonal directions. This has provided some interesting observations on the trends of this parameter in three dimensions in the supine position. The data provides a baseline against which other measurements of these profiles can be compared and it will be interesting to observe how consistently the variation described in this paper is found in future studies.

There were also differences in the distribution of air volume between the left and right lungs. The one dimensional profiles illustrated the lower values of FAVC for the left lung but the variations in the three orthogonal directions all showed similar patterns of variation for both lungs. The variation of FAVC with shell number again showed the consistently lower value for the left lung, but the relative difference was much greater for the inner shells resulting in a significantly lower central to peripheral ratio for the left lung. This is presumably as a result of the relatively higher proportion of major vessels in the reduced space volume of the lung envelope on the left side, due to the presence of the heart.

The results described in this paper on lung volume in the mid-tidal position are a by-product of a study carried out for a different purpose. For this reason not all aspects of the experimental design are optimised for lung volume measurement. Nevertheless, it is considered that useful novel results on the use of CT scanning to measure total and regional lung volume have been obtained. The number of subjects studied for example was not particularly optimised for this study. However from the repeat measurements on the 11 subjects studied, reasonably precise measures of average parameters of lung volume and repeatability have been calculated. In addition statistically significant conclusions on differences between parameters and correlations have been made. Comparison of the mid-tidal volume measurements obtained in the supine position with erect measurement of FRC was not ideal. In principle FRC would have ideally been carried out supine at the same time as the CT scan was being acquired, so that direct comparison of the measurements could be made.

Further work required on the spatial analysis of the distribution of volume is a description of the fractional air volume concentration by lobe and by segment. Since it is now possible to delineate lobes directly from CT images, and estimate the outline of the segments, lung volume parameters can be expressed per lobe and per segment. Initial work has shown that airway volume does vary regionally [35] and this will be the subject of future study.

The determination of regional lung volume at a particular point in the respiratory cycle using CT gives the possibility of looking at change in lung volume between different points of the cycle. The recent development of four dimensional CT (4DCT) enables dynamic CT imaging to be carried out over the respiratory cycle [36]. This would enable visualization of regional changes in volume, leading to high resolution quantitative ventilation imaging. This could potentially give important new information on lung physiology.

The potential of magnetic resonance imaging in providing information on lung volume needs also to be considered, as this provides three dimensional image data without incurring any risk due to ionizing radiation. Conventional proton MR does not provide images that are easily related to air volume, and so are unlikely to compete with CT for this purpose. However, measurements of regional lung volume would be clearly possible using hyperpolarised gas imaging [37]. If this were to become readily available then it could find an important role in this area.

## Conclusions

In conclusion a method of delineating the lung outline from CT images of the thorax has been described. This has been applied to low resolution CT scans obtained in control subjects at mean supine tidal volume. Average measurements of total lung volume and its regional distribution are described together with the inter-subject variability and the intra-subject variability of repeated measurements. The values obtained are reproducible and are in good agreement with previous results where such a comparison is possible. Fractional air volume concentration is shown to increase with lung size, a result which has important implications for the quantitative interpretation of measurements of lung density.

## Competing interests

This work was part of study (EudraCT # 2007-003563-43) which was sponsored by Air Liquide. JF acts as a consultant to Air Liquide. The University of Southampton has received funding from Astra Zeneca, Sweden for a study in which JF, JC and MB were involved.

## Authors’ contributions

JF designed the study, developed the image analysis software used in the study and drafted the manuscript. JC was the principal investigator of the overall project of which the current study was a part, and oversaw the clinical acquisition of the imaging data. CM performed the image analysis. MB and SM contributed to the design of the software. GC contributed to the study design. IK contributed to the study design and helped to draft the manuscript. All authors read and approved the final manuscript.

## Pre-publication history

The pre-publication history for this paper can be accessed here:

http://www.biomedcentral.com/1471-2342/14/25/prepub
